# The Sleep Expectation–Reality Gap: Exploring Discrepancies Between Perceived and Ideal Sleep Duration in Primary Care Patients

**DOI:** 10.3390/healthcare13060650

**Published:** 2025-03-17

**Authors:** Carlos De las Cuevas, María Segovia

**Affiliations:** 1 Department of Internal Medicine, Dermatology and Psychiatry, Universidad de La Laguna, 38200 San Cristóbal de La Laguna, Spain; 2 Instituto Universitario de Neurociencia (IUNE), Universidad de La Laguna, 38200 San Cristóbal de La Laguna, Spain; 3 School of Medicine, University of La Laguna, 38200 San Cristóbal de La Laguna, Spain; alu0101138701@ull.edu.es

**Keywords:** sleep discrepancy, perceived sleep, ideal sleep, insomnia, sleep expectations, primary care, sleep health

## Abstract

Objective: This study aimed to quantify the discrepancy between perceived and ideal sleep duration among primary care patients and identify demographic, lifestyle, and psychological factors associated with this expectation–reality gap. Methods: A cross-sectional study was conducted with 300 adult primary care patients, who completed a survey assessing demographics, sleep perceptions, and insomnia severity (Insomnia Severity Index, ISI). Sleep discrepancy was defined as the difference between perceived and ideal sleep duration. Statistical analyses included Wilcoxon signed-rank tests, Spearman’s correlations, and multiple linear regression to examine predictors of sleep discrepancy. Results: Participants reported a mean perceived sleep duration of 6.39 ± 1.36 h, significantly lower than their ideal sleep duration of 8.07 ± 0.75 h (*p* < 0.001). A significant sleep discrepancy was found in 81.3% of participants, while only 2.0% exceeded their perceived sleep needs. Higher ISI scores were strongly associated with greater sleep discrepancy (*r* = −0.476, *p* < 0.001). The regression model explained 27.7% of the variance (*p* < 0.001), with insomnia severity (β = −0.425, *p* < 0.001), higher BMI (β = −0.192, *p* < 0.001), cola drink consumption (β = 0.142, *p* = 0.009), and older age (β = 0.163, *p* = 0.002) as significant predictors. Gender, hypnotic medication use, and alcohol intake were non-significant. Conclusions: A substantial expectation–reality gap in sleep duration exists, linked to insomnia severity, older age, and lifestyle factors. Addressing maladaptive sleep expectations through cognitive–behavioral interventions in primary care may improve sleep satisfaction and reduce insomnia-related distress.

## 1. Introduction

Sleep is a fundamental component of cognitive, emotional, and physical health, yet dissatisfaction with sleep duration and quality is a prevalent issue among primary care patients [1,2]. Sleep complaints are often evaluated through objective measures such as polysomnography or actigraphy, but growing evidence suggests that subjective sleep perception plays a crucial role in sleep-related distress and health outcomes [3,4].

A well-established area of research has focused on sleep misperception—the discrepancy between subjective sleep reports and objective sleep measures [5]. Studies have shown that individuals with insomnia frequently underestimate their actual sleep duration, which is associated with heightened distress, cognitive hyperarousal, and increased risk for psychiatric comorbidities [3,6]. This subjective–objective sleep discrepancy is associated with poorer mental health outcomes and greater functional impairment [7,8]. However, while the relationship between perceived and objective sleep duration has been extensively studied, a critical yet underexplored issue is the expectation–reality gap in sleep—the discrepancy between perceived sleep duration and ideal sleep duration.

### 1.1. The Expectation–Reality Gap in Sleep: A Novel Framework

Expectations play a central role in subjective well-being and health perceptions. Research in pain management, mental health, and chronic disease outcomes has demonstrated that mismatched expectations can amplify psychological distress, symptom severity, and treatment dissatisfaction [9,10]. In the context of sleep, individuals often establish subjective sleep goals based on personal beliefs, societal norms, and public health recommendations [11,12]. This mismatch may be associated with sleep dissatisfaction, anxiety, and insomnia-like symptoms, even in the absence of objective sleep disturbances [13,14].

While previous studies have examined how sleep misperception affects distress and mental health, research has not adequately addressed how the gap between perceived and ideal sleep duration contributes to sleep-related dissatisfaction. This expectation–reality gap is likely involved in a reciprocal relationship with negative cognitive appraisals of sleep, sleep-related anxiety, behavioral maladaptation, and sleep disturbances—a pattern frequently observed in insomnia and psychophysiological sleep disorders [15,16].

### 1.2. Unmet Sleep Expectations in Primary Care

Primary care is a critical setting for addressing sleep dissatisfaction, as sleep complaints are among the most common concerns raised in general medical practice [17,18,19]. However, current approaches often focus on treating objective sleep deficits rather than addressing perceptual and psychological contributors to sleep dissatisfaction. Recognizing the expectation–reality gap as a unique contributor to sleep distress could shift clinical approaches toward cognitive and behavioral interventions that target sleep expectations rather than just sleep duration [20,21].

### 1.3. Study Objectives

To address this gap, this study aimed to quantify the discrepancy between perceived and ideal sleep duration in primary care patients and explored its associations with demographic and lifestyle factors. Specifically, we hypothesized:

- H1: There was a significant discrepancy between perceived sleep duration and the hours of sleep considered ideal by patients.
- H2: Demographic factors (e.g., age, sex) were associated with the magnitude of the expectation–reality gap in sleep.
- H3: Lifestyle habits (e.g., caffeine intake, alcohol consumption, medication use) were associated with sleep discrepancy severity.

By examining how individual characteristics and lifestyle factors contribute to sleep discrepancy, this study provided a novel psychological perspective on sleep dissatisfaction and offers potential avenues for behavioral interventions in primary care settings.

## 2. Material and Methods

### 2.1. Study Design and Participants

This was a cross-sectional observational study conducted among adults attending routine consultations in primary care centers.

- Inclusion Criteria: Adults aged ≥18 years, able to provide informed consent, and without diagnosed severe sleep disorders (e.g., obstructive sleep apnea, narcolepsy).
- Exclusion Criteria: Patients with severe cognitive impairments or those unable to complete the survey independently.

### 2.2. Data Collection

Participants completed a structured self-report, paper-and-pencil survey administered in person at the clinic during their routine primary care visits. The survey included the following sections:

- Demographics: Age, sex, weight, and height.
- Perceived Sleep Duration: “How many hours do you believe you sleep on an average night?”
- Ideal Sleep Duration: “How many hours do you think you should sleep each night to feel well-rested?”
- Sleep-Related Habits: Use of caffeine, alcohol, and medications.
- Insomnia Symptoms: Assessed using the Insomnia Severity Index (ISI).

### 2.3. Insomnia Severity Index (ISI)

Insomnia symptoms were assessed using the Insomnia Severity Index (ISI), a widely used self-report instrument designed to measure the severity of insomnia symptoms over the past two weeks. The ISI consists of 7 items that assess the severity of insomnia, including difficulties falling asleep, staying asleep, waking up too early, and the impact of insomnia on daily functioning. Each item is rated on a scale from 0 to 4, with a total score ranging from 0 to 28. The total score is categorized as follows: 0–7: No clinically significant insomnia; 8–14: Subthreshold (mild) insomnia; 15–21: Moderate insomnia; 22–28: Severe insomnia. These categories are used to classify the severity of insomnia and guide clinical assessment and treatment decisions.

### 2.4. Sleep Discrepancy Classification

Sleep discrepancy was assessed as the difference between actual sleep duration and self-reported ideal sleep duration. Participants were classified into three categories:

1. Sleep Deficit: Participants whose actual sleep duration was less than their perceived sleep need.
2. Adequate Sleep: Participants whose actual sleep duration-matched their perceived sleep needs.
3. Sleep Excess: Participants whose actual sleep duration exceeded their perceived sleep need.

This classification framework was used to analyze variations in sleep patterns and their potential impact on sleep health.

### 2.5. Statistical Analysis

All statistical analyses were conducted using SPSS (version 29) [22], with statistical significance set at *p* < 0.05. Data were first examined for normality (Shapiro–Wilk test) and homogeneity of variance (Levene’s test) to determine the appropriate statistical methods.

#### 2.5.1. Descriptive and Comparative Analyses

Descriptive statistics (means, standard deviations, and frequency distributions) were computed for demographic variables, lifestyle factors, perceived sleep duration, and ideal sleep duration. Differences between perceived and ideal sleep duration were assessed using the Wilcoxon signed-rank test, as data violated normality assumptions.

#### 2.5.2. Correlation and Predictive Modeling

Spearman’s rank correlation was used to examine associations between sleep discrepancy scores and variables such as stress levels (PSS), caffeine intake, alcohol consumption, and insomnia severity (ISI). Multiple linear regression models were conducted to identify predictors of sleep discrepancy, including age, sex, BMI, caffeine intake, alcohol use, medication use frequency, and ISI score.

## 3. Results

**Figure 1:**
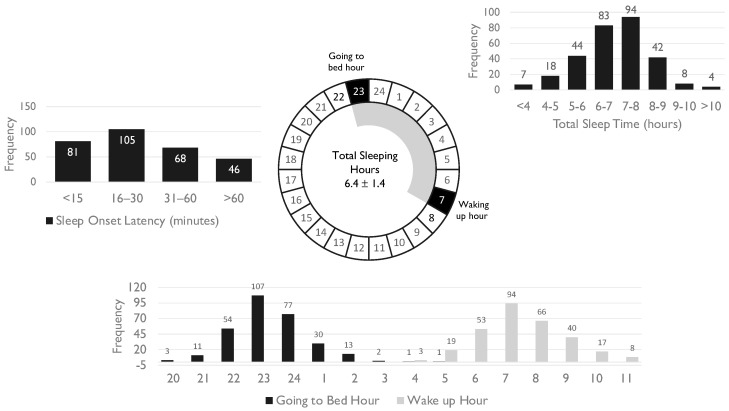
Median bedtime, wake-up time, and total sleep duration among primary care patients represented on a 24 h clock diagram, alongside bar graphs illustrating the distribution of sleep onset latency, total sleep duration, and going to bed and waking up hours.

Table 1 presents the demographic, clinical, and sleep-related characteristics of the study sample, including age, sex distribution, body mass index categories, sleep patterns, insomnia severity, medication use, lifestyle habits, and comorbid conditions. Figure 1 illustrates key sleep parameters of the study population, including sleep onset latency distribution, total sleep duration, bedtime and wake-up time frequencies, and a circular representation of average sleep duration.

### 3.1. Sample Characteristics

A total of 300 participants were included in the study, of whom 38.7% (n = 116) were male and 61.3% (n = 184) were female. The mean age of the sample was 49.73 years (SD = 17.61), ranging from 18 to 92 years. As shown in Table 1, the age distribution of the 300 primary care patients reveals a predominance of middle-aged and older adults, with over half of the sample (53.3%) aged 45 years or older. The largest group was late middle age (45–59 years, 29.0%), followed by older adults (60–74 years, 24.3%). The early middle age group (30–44 years) accounted for 23.3% of the sample, while young adults (18–29 years) represented 15.7%. The elderly population (≥75 years) comprised the smallest group (7.7%).

The reasons for consultation among the 300 primary care patients in the study were highly diverse, reflecting a wide range of somatic, psychological, chronic, and preventive health concerns. The most common consultations were for chronic conditions and follow-up visits, particularly hypertension (113 patients, 37.7%), diabetes (39 patients, 13.0%), dyslipidemia (74 patients, 24.7%), and respiratory diseases such as asthma and chronic obstructive pulmonary disease (COPD) (39 patients, 13.0%). Acute conditions were also frequently reported, with respiratory infections such as influenza (6.0%) and the common cold (1.7%), along with fever, sore throat, and cough. Musculoskeletal complaints—including back pain, joint pain, and lumbago—were another significant reason for consultation, as were gastrointestinal issues like acid reflux, abdominal pain, and constipation. Psychological and psychiatric concerns were present in a subset of patients, with anxiety-related complaints (4 patients, 1.3%), depression (3 patients, 1.0%), and panic attacks (1 patient, 0.3%) among the reasons for consultation. Additionally, lifestyle and behavioral health concerns, such as smoking cessation and weight-related issues, were reported in some cases.

### 3.2. Sleep Patterns

Participants’ reported bedtimes predominantly followed a late-night sleep schedule, with 35.7% going to bed at 23:00 and 25.7% at 24:00. Additionally, 14.3% of the sample reported going to bed between 24:00 and 2:00.

Regarding sleep onset latency, 35.0% of participants reported falling asleep within 16–30 min, while 27.0% fell asleep in less than 15 min. However, 15.3% of participants experienced prolonged sleep latency exceeding 60 min.

Wake-up time distribution exhibited a bimodal pattern, with 31.3% of participants waking up at 07:00 and 22.0% at 08:00. Only 6.3% reported waking up before 06:00, whereas 7.3% woke up after 09:00.

### 3.3. Sleep Duration and Perceived Optimal Sleep

The mean actual sleep duration reported was 6.39 h (SD = 1.36), with a minimum of 2 h and a maximum of 11 h. In contrast, the mean perceived optimal sleep duration was 8.07 h (SD = 0.75), with a median of 8 h.

### 3.4. Sleep Discrepancy Analysis

The Wilcoxon signed-rank test was conducted to compare participants’ perceived sleep duration and their ideal sleep duration. The results indicated a statistically significant difference (Z = −13.424, *p* < 0.001), suggesting that participants reported sleeping significantly fewer hours than they considered ideal. Descriptive statistics revealed that the mean actual sleep duration was 6.39 ± 1.36 h, while the mean perceived optimal sleep duration was 8.07 ± 0.75 h.

Further analysis of rank distributions showed that 244 out of 300 participants (81.3%) believed they should sleep more than they actually do, while only 6 participants (2.0%) reported sleeping more than their perceived need. The remaining 50 participants (16.7%) reported no discrepancy between actual and ideal sleep.

### 3.5. Use of Sleep Medication

Regarding the use of sleep medication, 73.7% of participants reported no use of sleep-inducing drugs in the past two weeks. However, 15.3% indicated taking sleep medication three or more times per week, while 5.7% reported using it once or twice per week.

### 3.6. Gender Differences in Sleep Latency, Duration, and Perceived Sleep Need

A chi-square test indicated no significant association between the time to fall asleep and gender (χ^2^(3) = 4.911, *p* = 0.178), suggesting that sleep latency distributions did not differ meaningfully between males and females.

The Kruskal–Wallis test performed to examine differences in actual sleep duration and perceived ideal sleep duration between males and females revealed a significant difference in actual sleep duration (H = 8.228, *p* = 0.004), indicating that females (mean rank = 161.57) reported sleeping significantly longer than males (mean rank = 132.94). Similarly, a highly significant difference was observed in perceived ideal sleep duration (H = 12.123, *p* < 0.001), where females (mean rank = 161.85) perceived their optimal sleep need to be significantly higher than males (mean rank = 132.50).

### 3.7. Age Differences in Sleep Latency, Duration, and Perceived Sleep Need

A Kruskal–Wallis test was conducted to assess whether age differs across sleep latency groups (<15 min, 16–30 min, 31–60 min, >60 min). The test revealed no significant differences in age between these groups (H = 1.291, df = 3, *p* = 0.731), suggesting that age does not significantly influence the time to fall asleep. Mean ranks were 143.56 for those falling asleep in less than 15 min, 156.48 for those taking 16–30 min, 153.39 for those taking 31–60 min, and 144.80 for those taking more than 60 min.

Spearman’s rank correlation was used to assess the relationship between age, actual sleep duration, and perceived ideal sleep duration due to the non-normal distribution of the data. The results indicated that age was not significantly correlated with actual sleep duration (ρ = 0.095, *p* = 0.101) or with perceived ideal sleep duration (ρ = −0.070, *p* = 0.224).

### 3.8. Insomnia Severity Index (ISI) Scores

The mean ISI total score in the sample was 11.12 (SD = 6.11), with scores ranging from 0 to 27. Based on established cutoff values, participants were classified into four insomnia severity categories: 33.0% (n = 99) reported no clinically significant insomnia (ISI = 0–7); 38.0% (n = 114) fell into the subthreshold (mild) insomnia category (ISI = 8–14); 24.0% (n = 72) were classified as having moderate insomnia (ISI = 15–21); and, 5.0% (n = 15) met criteria for severe insomnia (ISI = 22–28). Overall, 67.0% of participants reported some degree of insomnia symptoms (ISI ≥ 8), with 29.0% classified as moderate to severe insomnia (ISI ≥ 15).

### 3.9. Inferential Statistics

Relationship Between ISI Score and Sleep Discrepancy

A moderate negative correlation was found between ISI total score and sleep discrepancy (r = −0.476, *p* < 0.001), indicating that higher insomnia severity is associated with greater sleep deficit. Given the statistical significance and strength of this relationship, a linear regression analysis is recommended to further quantify the predictive value of ISI on sleep discrepancy. Additionally, a multiple regression model adjusting for age, BMI, gender, and medication use could help determine whether this relationship remains significant after controlling for potential confounders (Table 2). To assess the independence of predictors in the regression model, we conducted a multicollinearity check using variance inflation factors (VIFs). All predictors had VIF values well below 10, indicating no significant multicollinearity. Therefore, the predictors in the regression model were considered independent from each other, ensuring the validity of the results.

The regression analysis results indicate that the model explains 27.7% of the variance in sleep discrepancy (R^2^ = 0.277, adjusted R^2^ = 0.249), suggesting that insomnia severity and lifestyle factors contribute to the expectation–reality gap in sleep perception. The model is statistically significant (*p* < 0.001).

Among the individual predictors, insomnia severity (ISI Total; β = −0.425, *p* < 0.001) was the strongest factor, with higher ISI scores associated with greater sleep discrepancy. Additionally, higher BMI (β = −0.192, *p* < 0.001) and cola drink consumption (β = 0.142, *p* = 0.009) significantly predicted greater sleep discrepancy, indicating potential physiological or behavioral influences on sleep perception.

Age (β = 0.163, *p* = 0.002) was also a significant predictor, suggesting that older individuals tend to report a greater expectation–reality gap in sleep perception rather than a smaller one. This finding may reflect age-related changes in sleep expectations or sleep satisfaction, warranting further investigation.

Conversely, gender, hypnotic medication use, caffeine (coffee, tea, energy drinks), tobacco, and alcohol consumption were not significant predictors, indicating that these factors do not substantially contribute to subjective sleep misperception.

These findings underscore the complex psychological and lifestyle factors influencing sleep perception, emphasizing the need for targeted interventions in primary care to address cognitive distortions surrounding sleep expectations and improve sleep satisfaction.

## 4. Discussion

### 4.1. Sleep Discrepancy and Psychological Distress

Our study provides compelling evidence of a significant expectation–reality gap in sleep perception among primary care patients. Our results confirm that the majority of participants perceive their actual sleep duration as insufficient compared to their ideal sleep duration, with a statistically significant discrepancy of approximately 1.68 h. This mismatch aligns with prior research on sleep dissatisfaction and misperception [3,12] and underscores the psychological and behavioral implications of unmet sleep expectations.

One of the most notable findings is the strong association between sleep discrepancy and insomnia severity. Our regression analysis revealed that higher Insomnia Severity Index (ISI) scores were significantly associated with greater sleep discrepancy (β = −0.425, *p* < 0.001). This supports the cognitive hyperarousal model of insomnia, in which individuals with insomnia-like symptoms are more likely to perceive their sleep as inadequate, reinforcing psychological distress [6,7]. These findings suggest that interventions targeting cognitive distortions about sleep may be essential in mitigating sleep dissatisfaction and distress. Recent systematic reviews further support our results, highlighting cognitive factors, particularly maladaptive sleep beliefs, as critical mechanisms sustaining insomnia and potential therapeutic targets in cognitive–behavioral therapy for insomnia (CBT-I) [23,24].

Our results align with prior research demonstrating that greater subjective sleep discrepancy is associated with lower perceived restoration and well-being [25]. Their findings indicate that both shorter and longer subjective sleep durations are linked to increased sleep dissatisfaction, emphasizing the importance of individual perceptions in sleep-related distress. This further supports the need for interventions that address not only objective sleep duration but also subjective sleep expectations and misperceptions.

### 4.2. Cultural and Individual Variability in Sleep Perception

The concept of “enough sleep” is subjective and shaped by cultural norms, personal experiences, and biological factors. Some cultures emphasize productivity over rest, leading to an undervaluation of sleep duration [26]. Conversely, societies that prioritize well-being may promote longer sleep expectations.

Genetic and psychological factors also influence sleep perception. Chronotypes play a role, with evening types more likely to experience social jet lag, further distorting their subjective sleep estimates [27]. Additionally, stress and anxiety contribute to perceived sleep inadequacy, even when individuals achieve recommended sleep durations [26]. Busa et al. (2023) [28] recently demonstrated that lifestyle behaviors such as excessive smart device use and insufficient physical activity significantly contribute to poorer sleep quality and higher perceived stress, reinforcing our findings regarding behavioral impacts on sleep discrepancy.

### 4.3. Gender and Age Differences in Sleep Expectations

Our regression model found that age was significantly associated with greater sleep discrepancy (β = 0.163, *p* = 0.002), suggesting that older individuals tend to report a larger expectation–reality gap in sleep. This may reflect age-related changes in sleep quality, efficiency, and expectations, as older adults experience increased sleep fragmentation and shifts in circadian rhythms. Additionally, rigid sleep beliefs influenced by past patterns and societal norms could contribute to greater dissatisfaction with actual sleep duration. Future studies should explore age-stratified analyses and age-specific cognitive–behavioral interventions to address maladaptive sleep expectations in older adults.

In contrast, gender was not a significant predictor of sleep discrepancy (*p* = 0.885), despite prior research suggesting hormonal and social influences on sleep perception [29]. This finding implies that psychological and lifestyle factors may play a more dominant role in sleep misperception. While some studies suggest women report higher sleep dissatisfaction due to hormonal changes and stress sensitivity, our results indicate that these factors do not significantly impact the expectation–reality gap.

Overall, these findings highlight the importance of addressing sleep expectations in clinical practice, particularly in older adults, where perceived sleep deficits may be more distressing. Targeted interventions focusing on realistic sleep goals could help improve sleep satisfaction and reduce unnecessary sleep-related anxiety.

### 4.4. The Role of Lifestyle Factors in Sleep Perception

Among lifestyle variables, cola drink consumption emerged as a significant predictor of sleep discrepancy (β = 0.142, *p* = 0.009). This finding aligns with research indicating that caffeinated soft drinks may exacerbate sleep misperception and sleep dissatisfaction [30].

Conversely, coffee, tea, alcohol, hypnotic medication use, and tobacco consumption were not significant predictors in our model (*p* > 0.05). This suggests that while caffeine from cola beverages may influence sleep perceptions, other common lifestyle factors do not play a major role in shaping the expectation–reality gap in sleep.

Additionally, higher BMI was significantly associated with greater sleep discrepancy (β = −0.192, *p* < 0.001), reinforcing previous findings that obesity-related sleep disturbances, such as obstructive sleep apnea, may contribute to distorted sleep perceptions [30].

### 4.5. Clinical and Public Health Implications

The results of this study highlight the clinical relevance of sleep expectations in primary care settings. Physicians frequently encounter sleep complaints, yet interventions often focus on increasing sleep quantity rather than addressing maladaptive sleep beliefs. Given the strong association between insomnia severity and sleep discrepancy, targeted cognitive–behavioral interventions addressing sleep expectations and cognitive distortions may be crucial in reducing sleep dissatisfaction [15]. Recent meta-analyses emphasize the effectiveness of CBT-I in significantly improving sleep quality in younger populations, suggesting similar benefits could be achieved in primary care patients [31].

Additionally, public health campaigns should emphasize individual variability in sleep needs, rather than rigid recommendations such as the “8 h rule” [11]. Educating individuals to assess sleep quality and daytime functioning rather than focusing solely on sleep duration may reduce unnecessary sleep-related anxiety and improve overall sleep satisfaction.

## 5. Limitations

Despite the strengths of this study, several limitations should be acknowledged. First, the reliance on self-reported sleep data introduces the potential for recall bias and misestimation of actual sleep duration. Subjective sleep assessments are known to differ from objective measures, such as actigraphy or polysomnography, which may lead to discrepancies in reported sleep patterns [5]. Future research should incorporate objective sleep monitoring to validate these findings.

Second, the cross-sectional design of this study limits causal interpretations. While significant associations were observed between sleep discrepancy, insomnia severity, and demographic factors, the temporal direction of these relationships remains unclear. Longitudinal studies would help clarify whether sleep discrepancy contributes to increased insomnia severity over time or vice versa.

Third, the study sample consisted of primary care patients, which may not be fully representative of the general population. Individuals seeking medical care may have a higher prevalence of sleep disturbances or health conditions that influence sleep perceptions. Future studies should examine sleep discrepancy in broader community-based samples to enhance generalizability.

Lastly, while we explored lifestyle factors such as caffeine and alcohol use, additional psychosocial variables, including stress levels, occupational demands, and mental health status, were not extensively analyzed. These factors may play a crucial role in shaping sleep perceptions and should be considered in future investigations.

## 6. Conclusions

This study contributes to the growing body of literature on subjective sleep perception by identifying a significant expectation–reality gap in sleep among primary care patients. Our findings emphasize that the perceived mismatch between actual and ideal sleep duration plays a crucial role in shaping sleep-related well-being, expanding prior research that primarily focused on the discrepancy between subjective and objective sleep duration. This highlights the psychological and behavioral implications of unmet sleep expectations, reinforcing the need for targeted cognitive interventions to address sleep dissatisfaction.

The strong association between sleep discrepancy and insomnia severity underscores the importance of psychological approaches that focus not only on sleep quantity but also on cognitive appraisals of sleep adequacy. Furthermore, gender differences and BMI-related sleep perceptions highlight the complexity of sleep misperception and its broader implications for health. These insights provide a novel framework for addressing sleep dissatisfaction in primary care settings, advocating for interventions that shift the focus from rigid sleep duration goals toward individualized and realistic sleep expectations.

Future research should explore the mechanisms underlying these discrepancies and evaluate intervention strategies that can effectively align sleep perceptions with actual sleep patterns, ultimately improving sleep health and overall well-being. By addressing both the physiological and cognitive aspects of sleep perception, clinicians and researchers can develop more comprehensive strategies to mitigate sleep dissatisfaction and its associated health risks.

## Figures and Tables

**Table 1:** Demographic, clinical, and sleep characteristics of the study sample.

Variable	Mean ± SD (or %)
Demographic Data	
Age (years)	49.7 ± 17.6
Young adults (18–29 years)	15.7% (47/300)
Early middle age (30–44 years)	23.3% (70/300)
Late middle age (45–59 years)	29.0% (87/300)
Older adults (60–74 years)	24.3% (73/300)
Elderly (≥75 years)	7.7% (23/300)
Sex	
Male	38.7% (116/300)
Female	61.3% (184/300)
Weight (kg)	74 ± 17
Height (cm)	168 ± 10
Body mass index	
Underweight (<18.5)	2.3% (7/300)
Normal weight (18.5–24.9)	37.7% (113/300)
Overweight (25.0–29.9)	43.7% (131/300)
Obese class I (30.0–34.9)	10.0% (30/300)
Obese class II (35.0–39.9)	3.7% (11/300)
Obese class III (≥40.0)	2.3% (7/300)
Sleep Patterns	
Bedtime (median time)	23.00
Sleep onset latency	
<15 min	27.0% (81/300)
16–30 min	35.0% (105/300)
31–60 min	22.7% (68/300)
>60 min	15.3% (46/300)
Wake-up time (median time)	7.00
Actual sleep hours per night	6.4 ± 1.4
Perceived optimal sleep hours	8.1 ± 0.8
Insomnia	
No clinically significant insomnia	33% (99/300)
Subthreshold (mild) insomnia	38% (114/300)
Moderate insomnia	24% (72/300)
Severe insomnia	5% (15/300)
Medication use	
Never	73.7% (221/300)
Less than once a week	5.3% (16/300)
Once or twice a week	5.7% (17/300)
Three or more times a week	15.3% (46/300)
Lifestyle Habits	
Coffee (Yes)	73.3% (220/300)
Tea (Yes)	19% (57/300)
Cola drinks (Yes)	21.3% (64/300)
Energy drinks (Yes)	5.3% (16/300)
Cigarettes (Yes)	25.3% (76/300)
Alcohol (Yes)	46.7% (140/300)
Current Treatments	
Hypertension	37.7% (113/300)
Diabetes	13.0% (39/300)
Dyslipidemia	24.7% (74/300)
Asthma	6.3% (19/300)
COPD	6.7% (20/300)

**Table 2:** Multiple linear regression results predicting sleep discrepancy.

Model					95% CI	*p* Value
		B	Std. Error	Beta		
1	(Constant)	0.013	0.467		[−0.91, 0.94]	0.978
	Gender	−0.022	0.149	−0.008	[−0.34, 0.30]	0.885
	Age	0.013	0.004	0.163	[0.005, 0.021]	0.002
	ISI total	−0.096	0.013	−0.425	[−0.12, −0.07]	<0.001
	Body mass index	−0.053	0.015	−0.192	[−0.08, −0.02]	<0.001
	Hypnotics	−0.054	0.171	−0.017	[−0.39, 0.28]	0.755
	Coffee	0.046	0.158	0.015	[−0.27, 0.36]	0.769
	Tea	0.103	0.181	0.029	[−0.25, 0.46]	0.569
	Cola drinks	0.478	0.182	0.142	[0.12, 0.84]	0.009
	Energy drinks	0.068	0.321	0.011	[−0.56, 0.70]	0.831
	Tobacco	−0.247	0.169	−0.078	[−0.58, 0.08]	0.145
	Alcohol	0.111	0.146	0.040	[−0.18, 0.40]	0.449

Model Summary: Model 1: R = 0.526, R^2^ = 0.277, adjusted R^2^ = 0.249, standard error of the estimate = 1.19483; F(11, 288) = 9.543, *p* < 0.001; effect sizes: Cohen’s f^2^ = 0.38 (moderate effect); Durbin–Watson = 1.944. Predictors: (constant), alcohol, coffee, cola drinks, hypnotics, tea, age, gender, energy drinks, body mass index, tobacco, ISI total. Dependent variable: discrepancy. B represents unstandardized coefficients; Beta represents standardized coefficients.

## Data Availability

Dataset available on request from the authors.

## References

[1] Lallukka T., Sivertsen B., Kronholm E., Bin Y.S., Øverland S., Glozier N. (2018). Association of Sleep Duration and Sleep Quality with the Physical, Social, and Emotional Functioning among Australian Adults. Sleep Health.

[2] Baranwal N., Yu P.K., Siegel N.S. (2023). Sleep Physiology, Pathophysiology, and Sleep Hygiene. Prog. Cardiovasc. Dis..

[3] Rezaie L., Fobian A.D., McCall W.V., Khazaie H. (2018). Paradoxical Insomnia and Subjective-Objective Sleep Discrepancy: A Review. Sleep Med. Rev..

[4] Chattu V.K., Manzar M.D., Kumary S., Burman D., Spence D.W., Pandi-Perumal S.R. (2018). The Global Problem of Insufficient Sleep and Its Serious Public Health Implications. Healthcare.

[5] Ma Y., Goldstein M.R., Davis R.B., Yeh G.Y. (2021). Profile of Subjective-Objective Sleep Discrepancy in Patients with Insomnia and Sleep Apnea. J. Clin. Sleep Med..

[6] Harvey A.G., Stinson K., Whitaker K.L., Moskovitz D., Virk H. (2008). The Subjective Meaning of Sleep Quality: A Comparison of Individuals with and without Insomnia. Sleep.

[7] Riemann D., Spiegelhalder K., Feige B., Voderholzer U., Berger M., Perlis M., Nissen C. (2010). The Hyperarousal Model of Insomnia: A Review of the Concept and Its Evidence. Sleep Med. Rev..

[8] Altena E., Baglioni C., Espie C.A., Ellis J., Gavriloff D., Holzinger B., Schlarb A., Frase L., Jernelöv S., Riemann D. (2020). Dealing with Sleep Problems during Home Confinement Due to the COVID-19 Outbreak: Practical Recommendations from a Task Force of the European CBT-I Academy. J. Sleep Res..

[9] de Lange F.P., Heilbron M., Kok P. (2018). How Do Expectations Shape Perception?. Trends Cogn. Sci..

[10] Kirkbride J.B., Anglin D.M., Colman I., Dykxhoorn J., Jones P.B., Patalay P., Pitman A., Soneson E., Steare T., Wright T. (2024). The Social Determinants of Mental Health and Disorder: Evidence, Prevention and Recommendations. World Psychiatry.

[11] Hirshkowitz M., Whiton K., Albert S.M., Alessi C., Bruni O., DonCarlos L., Hazen N., Herman J., Adams Hillard P.J., Katz E.S. (2015). National Sleep Foundation’s Updated Sleep Duration Recommendations: Final Report. Sleep Health.

[12] Trimmel K., Eder H.G., Böck M., Stefanic-Kejik A., Klösch G., Seidel S. (2021). The (Mis)Perception of Sleep: Factors Influencing the Discrepancy between Self-Reported and Objective Sleep Parameters. J. Clin. Sleep Med..

[13] Draycott S., Dabbs A. (1998). Cognitive Dissonance. 1: An Overview of the Literature and Its Integration into Theory and Practice in Clinical Psychology. Br. J. Clin. Psychol..

[14] Steptoe A., O’Donnell K., Marmot M., Wardle J. (2008). Positive Affect, Psychological Well-Being, and Good Sleep. J. Psychosom. Res..

[15] Morin C.M., Vallières A., Ivers H. (2007). Dysfunctional beliefs and attitudes about sleep (DBAS): Validation of a brief version (DBAS-16). Sleep.

[16] Levenson J.C., Kay D.B., Buysse D.J. (2015). The Pathophysiology of Insomnia. Chest.

[17] Senthilvel E., Auckley D., Dasarathy J. (2011). Evaluation of Sleep Disorders in the Primary Care Setting: History Taking Compared to Questionnaires. J. Clin. Sleep Med..

[18] Torrens Darder I., Argüelles-Vázquez R., Lorente-Montalvo P., Torrens-Darder M.D.M., Esteva M. (2021). Primary Care Is the Frontline for Help-Seeking Insomnia Patients. Eur. J. Gen. Pract..

[19] Oster A., Wiking E., Nilsson G.H., Olsson C.B. (2024). Patients’ Expectations of Primary Health Care from Both Patients’ and Physicians’ Perspectives: A Questionnaire Study with a Qualitative Approach. BMC Prim. Care.

[20] Irish L.A., Kline C.E., Gunn H.E., Buysse D.J., Hall M.H. (2015). The Role of Sleep Hygiene in Promoting Public Health: A Review of Empirical Evidence. Sleep Med. Rev..

[21] Chow C.M. (2020). Sleep and Wellbeing, Now and in the Future. Int. J. Environ. Res. Public Health.

[22] IBM Corp. (2023). IBM SPSS Statistics for Windows.

[23] Tang N.K.Y., Saconi B., Jansson-Fröjmark M., Ong J.C., Carney C.E. (2023). Cognitive factors and processes in models of insomnia: A systematic review. J Sleep Res..

[24] Nielson S.A., Perez E., Soto P., Boyle J.T., Dzierzewski J.M. (2023). Challenging beliefs for quality sleep: A systematic review of maladaptive sleep beliefs and treatment outcomes following cognitive behavioral therapy for insomnia. Sleep Med Rev..

[25] Kalak N., Brand S., Beck J., Holsboer-Trachsler E., Wollmer M.A. (2015). Association between subjective actual sleep duration, subjective sleep need, age, body mass index, and gender in a large sample of young adults. Neuropsychiatr Dis Treat..

[26] Grandner M.A. (2017). Sleep, Health, and Society. Sleep Med. Clin..

[27] Montaruli A., Castelli L., Mulè A., Scurati R., Esposito F., Galasso L., Roveda E. (2021). Biological Rhythm and Chronotype: New Perspectives in Health. Biomolecules.

[28] Busa F., Csima M.P., Márton J.A., Rozmann N., Pandur A.A., Ferkai L.A., Deutsch K., Kovács Á., Sipos D. (2023). Sleep Quality and Perceived Stress among Health Science Students during Online Education-A Single Institution Study. Healthcare.

[29] Oh C.M., Kim H.Y., Na H.K., Cho K.H., Chu M.K. (2019). The Effect of Anxiety and Depression on Sleep Quality of Individuals with High Risk for Insomnia: A Population-Based Study. Front. Neurol..

[30] Watson N.F., Harden K.P., Buchwald D., Vitiello M.V., Pack A.I., Weigle D.S., Goldberg J. (2012). Sleep Duration and Body Mass Index in Twins: A Gene-Environment Interaction. Sleep.

[31] Tadros M., Newby J.M., Li S., Werner-Seidler A. (2025). A systematic review and meta-analysis of psychological treatments to improve sleep quality in university students. PLoS ONE.

